# A Rare Case of Primary Meningococcal Myopericarditis in a 71-Year-Old Male

**DOI:** 10.1155/2016/1297869

**Published:** 2016-11-28

**Authors:** Odilia I. Woudstra, Gerard J. J. Boink, Jacobus A. Winkelman, Ron van Stralen

**Affiliations:** ^1^Department of Cardiology, Tergooi Ziekenhuis, Blaricum, Netherlands; ^2^Heart Center, Department of Cardiology, Academic Medical Center, Amsterdam, Netherlands; ^3^Heart Center, Department of Cardiothoracic Surgery, Academic Medical Center, Amsterdam, Netherlands

## Abstract

We describe a case of primary meningococcal C pericarditis with myocardial involvement in a 71-year-old male that is thus far the oldest patient with isolated meningococcal pericardial disease and only the third patient with primary meningococcal myopericarditis described in English literature. Our patient was successfully treated by full sternotomy and surgical drainage combined with intravenous ceftriaxone. Mild symptoms unresponsive to anti-inflammatory treatment and leukocytosis may guide clinicians towards the correct diagnosis. It is important to recognize this cause of pericarditis as the relatively mild clinical presentation may rapidly progress into tamponade and right-sided heart failure.

## 1. Introduction

Acute pericarditis is a common disease with many possible causes. Idiopathic etiology, that is, viral or immune mediated, is the most common cause, at least in the developed countries. Since the introduction of antibiotics, bacteria are only rarely (<1% of all cases) the cause of pericarditis [1, 2], causing purulent pericardial effusion. In this report we describe a case of primary meningococcal myopericarditis (defined as pericarditis with myocardial involvement), which illustrates the importance of early recognition given the potential for rapid disease progression.

## 2. Case Presentation

**Figure 1:**
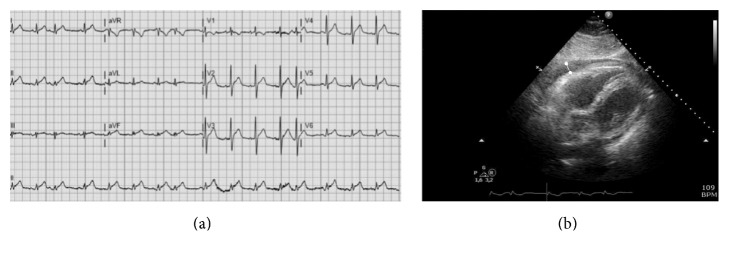
ECG and echocardiography at admission. (a) Twelve-lead ECG during admission shows sinus rhythm and premature atrial contractions in combination with diffuse ST-elevation, typical for acute pericarditis. (b) Ultrasound studies indicated moderate (<20 mm) pericardial effusion, here shown on the subcostal view and indicated with the white marker.

A previously healthy 71-year-old male presented to the cardiac care unit with intermittent chest pain over the previous 24 hours and worsening of discomfort and pain by lying on his left side. Other symptoms consisted of pleuritic pain between the shoulders, a sore throat, and several days of low-grade fever. On presentation, he was not acutely ill despite a temperature of 38.8°C. Clinical examination of heart and lungs showed no abnormalities. He had one painfully enlarged lymph node on the right side of his neck. ECG showed sinus rhythm with normal axis and conduction times and widespread ST-elevation (Figure 1(a)). Laboratory findings showed troponin-T of 22 ng/l (normal value < 14 ng/l), CRP of 310 mg/l, and leukocytosis (21.1 × 10*e*9/*l*) with a left shift (19 × 10*e*9/*l* granulocytes). A chest X-ray showed no abnormalities.

We diagnosed our patient with myopericarditis and based on the epidemiology we considered an idiopathic or reactive origin most likely. We hypothesized that a bacterial infection of the throat could explain the infectious parameters. A bacterial myopericarditis was also considered, but anticipated less likely given the absence of acute illness. With this differential diagnosis we took blood cultures and started treatment with amoxicillin and clavulanic acid (1000/200 mg 3 times daily) in combination with acetylsalicylic acid (600 mg 4 times daily) and pantoprazole.

On the second day of admission, the ear, nose, and throat doctor was consulted, who excluded pharyngeal abscess as alternative cause for the elevated infectious parameters. A cardiac ultrasound study showed normal heart function and moderate (<20 mm) pericardial effusion (Figure 1(b)). During admission to our ward, chest pain remained unchanged and inflammatory parameters increased to CRP 495 mg/l and leukocytes of 27 × 10*e*9/*l*. On the third day, 2 blood cultures grew positive of* Neisseria meningitidis *serotype C (subtype P 1.5, 2, F3-3) sensitive for amoxicillin, rifampicin, and ceftriaxone, and antibiotics were switched to ceftriaxone (2 grams i.v. once daily). The patient's wife was prophylactically treated with azithromycin (500 mg once daily for 3 days).

**Figure 2:**
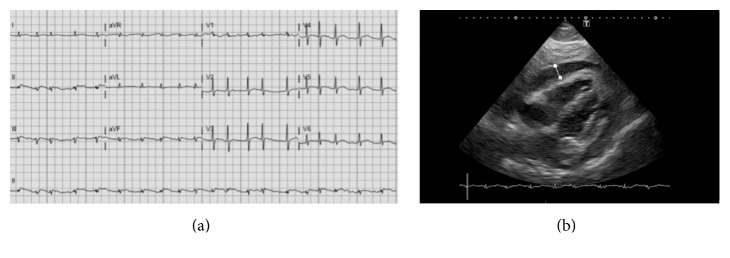
ECG and echocardiography on day 4. (a) Twelve-lead ECG during the fourth day of admission shows atrial fibrillation and microvoltages, indicative of increased pericardial effusion. (b) Ultrasound studies confirmed the presence of large (>20 mm) pericardial effusion, here shown on the subcostal view and indicated with the white marker.

On the fourth day, our patient had developed paroxysmal atrial fibrillation, and ECG voltages were decreased compared to admission (Figure 2(a)). A repeat ultrasound showed a substantial increase in pericardial fluid to large (>20 mm) pericardial effusion, with dense structures within the fluid (Figure 2(b)). Furthermore, it showed diffuse left ventricular hypokinesis with a decreased ejection fraction of ±30%, a mild right ventricular diastolic collapse, and a fixed and unreactive inferior vena cava. Importantly, our patient had no fever and did not show other clinical signs of disease progression.

**Figure 3:**
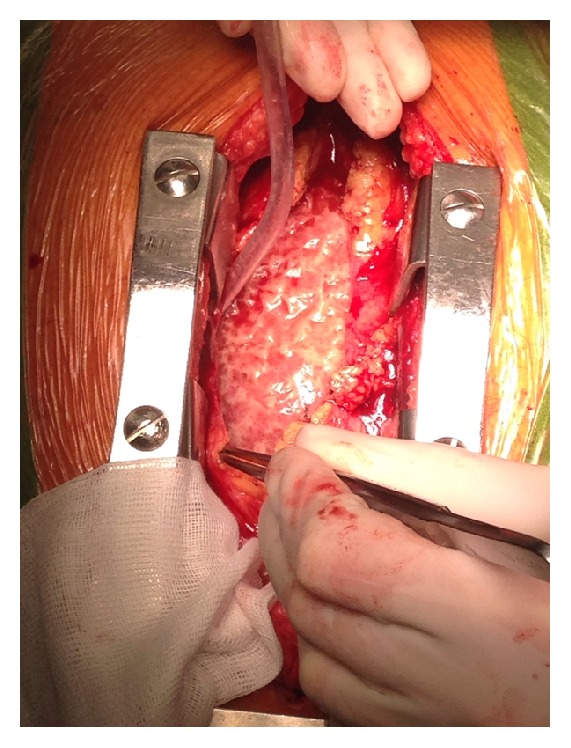
Bread and butter appearance upon opening the pericardium. The “bread and butter” appearance seen upon separating the visceral and parietal surfaces of the pericardium during surgery is typical for fibrinous pericarditis.

By all means, the ECG and ultrasound studies clearly indicated early cardiac tamponade and our patient was immediately transferred to a tertiary referral hospital. Sufficient drainage via pericardiocentesis or a pericardial window was considered unlikely given the dense aspect of the pericardial effusion on echocardiography. Therefore, the patient immediately underwent complete pericardiotomy via sternotomy to remove all debris. Upon opening the pericardium, a typical bread and butter appearance was noted (Figure 3), after which 1100 milliliters of thick purulent fluid was removed and chest tubes were placed to drain the pericardial space. Gram stains were positive for gram-negative diplococcus. Cultures did not grow bacteria, presumably as a result of antibiotic treatment. The patient returned to our hospital after the removal of pericardial drains. Postoperatively he had signs of right-sided decompensation and persisting atrial fibrillation, for which he was treated with furosemide, metoprolol, and acenocoumarol. After completing two weeks of ceftriaxone, infectious parameters were normalized, ultrasound showed no return of pericardial effusion, and the patient was discharged in good health. After three months of follow-up, left ventricle ejection fraction was completely recovered and there were no signs of constrictive pericarditis.

## 3. Discussion

Meningococci account for approximately 6% of all cases of purulent pericarditis [3], being the fourth most common bacterial cause following staphylococci, pneumococci, and streptococci [4]. Meningococcal pericarditis can be classified into three types of disease: (1) pericarditis in disseminated meningococcal disease, (2) primary (isolated) meningococcal pericarditis, and (3) reactive meningococcal pericarditis, which causes serous and sterile pericardial effusion in the postinfectious course of meningococcal disease after successful treatment with antibiotics [5].

Primary (isolated) meningococcal pericarditis (PMP) is defined as pericarditis with positive blood or pericardial fluid cultures without signs of meningeal involvement, meningococcemia, or involvement of other organs [5–7]. Approximately 30 cases of primary meningococcal pericarditis have been described in English literature, affecting mostly teenagers and young adults. Like our patient, they presented with chest pain, fever, leukocytosis, and a 2-day history of symptoms [6]. Only two cases of primary meningococcal myopericarditis have been reported [8, 9]. To the best of our knowledge, our patient is the oldest in whom isolated meningococcal pericardial disease has been described.

A large proportion of the population is nasopharyngeal carrier of* Neisseria meningitidis*. Some hypothesize that a low-grade infection of the bloodstream brings the bacteria to the pericardium [5, 10]. The immune system effectively eliminates these bacteria from the bloodstream but is less effective in clearing the pericardial space. The sore throat of our patient may have been caused by pharyngitis (throat cultures were positive for* Pantoea agglomerans*), which could have eased the way for residing meningococci to enter the bloodstream. Blaser et al. [3] showed that meningococcus type C is the most common serotype causing PMP, accounting for up to 88% of all PMP cases. This is more than expected, since this serotype is less prevalent in meningitis and meningococcemia (22%).

Like our patient, patients with PMP may not be severely ill on presentation [11]. This complicates differentiating PMP from viral pericarditis. This differentiation is important, because cardiac tamponade occurs in up to 88% of cases of PMP [6]. The lack of reaction to anti-inflammatory treatment combined with leukocytosis may point to PMP. Treatment of PMP consists of intravenous antibiotics, in most cases combined with drainage of the pericardial cavity [3]. Contrary to other causes of purulent pericarditis, PMP has good prognosis when timely diagnosed and treated. All patients previously described in English literature have survived [6, 8, 11].

In conclusion, primary meningococcal pericarditis is a very rare disease, especially when there is myocardial involvement. It initially presents with symptoms similar to viral pericarditis but can rapidly progress into cardiac tamponade. Leukocytosis and no reaction to anti-inflammatory treatment should trigger clinicians to think of meningococcal and other bacterial forms of pericarditis.

## References

[1] Imazio M., Spodick D. H., Brucato A., Trinchero R., Adler Y. (2010). Controversial issues in the management of pericardial diseases. *Circulation*.

[2] Imazio M., Gaita F. (2015). Diagnosis and treatment of pericarditis. *Heart*.

[3] Blaser M. J., Reingold A. L., Alsever R. N., Hightower A. (1984). Primary meningococcal pericarditis: a disease of adults associated with serogroup C *Neisseria meningitidis*. *Reviews of Infectious Diseases*.

[4] Sagristà-Sauleda J., Barrabés J. A., Permanyer-Miralda G., Soler-Soler J. (1993). Purulent pericarditis: review of a 20-year experience in a general hospital. *Journal of the American College of Cardiology*.

[5] Finkelstein Y., Adler Y., Nussinovitch M., Varsano I., Amir J. (1997). A new classification for pericarditis associated with meningococcal infection. *European Journal of Pediatrics*.

[6] Baevsky R. H. (1999). Primary meningococcal pericarditis. *Clinical Infectious Diseases*.

[7] Falcão S. N. R. S., Tsutsui J. M., Ramires F. J. (2007). The role of echocardiography in diagnosis and management of isolated meningococcal pericarditis. *Echocardiography*.

[8] Nkosi J., Thakrar A., Kumar K. (2009). Meningococcal serotype Y myopericarditis. *Diagnostic Microbiology and Infectious Disease*.

[9] Ejlertsen T., Vesterlund T., Schmidt E. B. (1988). Myopericarditis with cardiac tamponade caused by Neisseria meningitidis serogroup W135. *European Journal of Clinical Microbiology & Infectious Diseases*.

[10] Hardy D. J., Bartholomew W. R., Amsterdam D. (1986). Pathophysiology of primary meningococcal pericarditis associated with *Neisseria meningitidis* group C. a case report and review of the literature. *Diagnostic Microbiology and Infectious Disease*.

[11] Zeidan A., Tariq S., Faltas B., Urban M., McGrody K. (2008). A case of primary meningococcal pericarditis caused by Neisseria meningitidis serotype Y with rapid evolution into cardiac tamponade. *Journal of General Internal Medicine*.

